# Myocarditis Following COVID-19 Vaccine Use: Can It Play a Role for Conditioning Immunization Schedules?

**DOI:** 10.3389/fimmu.2022.915580

**Published:** 2022-06-27

**Authors:** Susanna Esposito, Caterina Caminiti, Rosanna Giordano, Alberto Argentiero, Greta Ramundo, Nicola Principi

**Affiliations:** ^1^ Pediatric Clinic, Department of Medicine and Surgery, University Hospital of Parma, Parma, Italy; ^2^ Research and Innovation Unit, University Hospital of Parma, Parma, Italy; ^3^ Department of Public Health, AUSL Parma, Parma, Italy; ^4^ Università degli Studi di Milano, Milan, Italy

**Keywords:** COVID-19, COVID-19 vaccine, myocarditis, heart, vaccine safety

## Abstract

Myocarditis (MYO) is a relatively uncommon inflammatory disease that involves the heart muscle. It can be a very severe disease as it can lead to the development of acute or chronic heart failure and, in a not marginal number of cases, to death. Most of the cases are diagnosed in healthy people younger than 30 years of age. Moreover, males are affected about twice as much as females. Viruses are among the most common causes of MYO, but how viral infection can lead to MYO development is not precisely defined. After COVID-19 pandemic declaration, incidence rate of MYO has significantly increased worldwide because of the SARS-CoV-2 infection. After the introduction of anti-COVID-19 vaccines, reports of post-immunization MYO have emerged, suggesting that a further cause of MYO together with the SARS-CoV-2 infection could increase the risk of heart damage during pandemic. Main aim of this study is to discuss present knowledge regarding etiopathogenesis and clinical findings of MYO associated with COVID-19 vaccine administration and whether the risk of this adverse events can modify the initially suggested recommendation for the use of COVID-19 vaccines in pediatric age. Literature analysis showed that MYO is an adverse event that can follow the COVID-19 immunization with mRNA vaccines in few persons, particularly young adults, adolescents, and older children. It is generally a mild disease that should not modify the present recommendations for immunization with the authorized COVID-19 mRNA vaccines. Despite this, further studies are needed to evaluate presently undefined aspects of MYO development after COVID-19 vaccine administration and reduce the risk of development of this kind of vaccine complication. Together with a better definition of the true incidence of MYO and the exact role of the various factors in conditioning incidence variations, it is essential to establish long-term evolution of acute COVID-19 related MYO.

## Introduction

Myocarditis (MYO), a condition frequently associated with pericarditis causing the so called myopericarditis, is an inflammatory disease that involves the heart muscle (1). It can be a very severe disease as it can lead to the development of acute or chronic heart failure and, in a not marginal number of cases, to death. MYO can affect anyone, including young adults, children and infants. Most of the cases are diagnosed in healthy people younger than 30 years of age. Moreover, males are affected about twice as much as females (1). Autoimmune, neoplastic, metabolic risk factors and specific drug use have been associated with MYO development (1). However, viruses are among the most common causes of MYO, with cardiotropic viruses, such as adenoviruses and enteroviruses, the most frequently identified agents (2–7). Moreover, cases associated with infection due to herpesvirus 6, human immunodeficiency virus, and respiratory syncytial virus have been reported (8).

How viral infection can lead to MYO development is not precisely defined. It is supposed that viruses can cause a direct myocardial injury and an exacerbated and uncontrolled immune response, despite leading to the clearance of the virus-infected myocytes, can play a fundamental role in causing a further increase in myocardial injury or necrosis (9). Although the true incidence of this disease is not known as several acute cases are subclinical and are not diagnosed (10), MYO is considered a relatively uncommon disease. Several epidemiological studies enrolling hospitalized MYO cases before COVID-19 pandemic development have shown that the global incidence of this disease was approximately 1–10 cases per 100,000 people per year (11), with the highest values in adolescents and young adults (12). However, in 2020 after COVID-19 pandemic declaration, incidence rate of MYO has significantly increased worldwide (13). The main factor for this increase has been identified in the SARS-CoV-2 infection itself. Data collected in hospitalized adult patients with COVID-19 have shown that cardiac problems including simple elevation of cardiac markers could be detected in up to 30% of cases and that even higher prevalence of signs of heart damage could be identified in patients with previous heart disease (14–17). A study carried out in the USA in which hospital-based administration data were used and incidence of MYO in 2019 and 2020 was compared showed that the occurrence of MYO was 42.3% (95% confidence interval [CI] 14.1–17.2) higher after pandemic declaration than before (18). During March 2020–January 2021,the risk for MYO was 0.146% among patients diagnosed with COVID-19 and 0.009% among patients without, leading to the conclusion that the mean risk for MYO in patients with COVID-19 was nearly 16 times higher, with the highest values in children, older adults, and males. Risk ratios ranged from approximately 7.0 for patients aged 16–39 years to >30.0 for patients aged <16 years or ≥75 years. However, after the introduction of anti-COVID-19 vaccines, despite the lack of an observed risk from preauthorization clinical trials’ data (19–21), several reports of post-immunization MYO have emerged, suggesting that a further cause of MYO together with the SARS-CoV-2 infection could increase the risk of heart inflammation during pandemic.

Main aim of this review is to discuss present knowledge regarding etiopathogenesis and clinical findings of MYO associated with COVID-19 vaccine administration and whether the risk of this adverse events can modify the initially suggested recommendation for the use of COVID-19 vaccines in pediatric age. The literature search was performed on the PubMed database, with a selection of English-language articles published from 2020. Key search terms were: “myocarditis” OR “myopericarditis” AND “COVID-19 vaccine” OR “COVID-19 vaccination” AND “children” OR “adolescent” OR “pediatric” OR “paediatric” were used.

## COVID-19 Vaccines and Myocarditis

Available studies, despite with significant differences, indicate that in the general population the risk of MYO development after COVID-19 vaccine administration exists, but it is lower than that due to SARS-CoV-2 infection, although higher than that reported in healthy patients before the pandemic (14–21). Unfortunately, studies that have monitored MYO development after COVID-19 vaccination cannot be compared as they significantly differ for several factors, including the different criteria used to define MYO. Endomyocardial biopsy, that is the most reliable method to diagnose MYO through the evidence of an inflammatory cell infiltration with or without corresponding myocardial damage was not performed in most of suspected MYO cases, and hyperdiagnosis or hypodiagnosis can be supposed. Moreover, characteristics of vaccinated subjects significantly differ from study to study for type of administered vaccine, sex and age of vaccinated subjects, and length of follow-up after immunization. This can explain why absolute values of incidence, time of development of heart manifestations, role of age and sex in favoring MYO have been differently reported in the various studies. However, all researches agree that MYO development is strictly related to the use of mRNA vaccines and seems to occur more frequently in association with the Moderna mRNA-1273 vaccine rather than after the Pfizer-BioNTech BNT162b2 vaccine (22–24). Moreover, MYO has been systematically reported significantly more frequently after the second vaccine dose in males and in people younger than 30 years of age (22–24). Finally, it was generally diagnosed early after immunization, within the first week both after the first and the second vaccine dose (22–24). The importance of mRNA vaccines as cause of MYO is clearly evidenced by a great number of case reports, several retrospective studies and by a case-control study analyzing carditis risk associated with 2 different COVID-19 vaccine technologies (12, 22–27). In most of the cases, only mRNA vaccines were found associated with MYO development. Moreover, although retrospective evaluations of adverse events following COVID-19 vaccine administration have reported MYO even after Vaxzevria, the adenoviral vector vaccine made by AstraZeneca, cases were not reported more frequently than the background rate (28). Finally, a study comparing risk of MYO in people aged 12 years or older receiving the Pfizer-BioNTech vaccine or the inactivated CoronaVAC vaccine showed that subjects receiving the mRNA vaccine had higher odds of carditis (adjusted odds ratio [aOR] 3.57; 95% CI 1.93-6.60] than unvaccinated persons, whereas no true association with MYO could be demonstrated for those vaccinated with CoronaVac (29).

The USA Vaccine Safety Datalink analysis has calculated that the adjusted rate ratio (aRR) of MYO/pericarditis development in the period 1-21 days after immunization with one of the two mRNA vaccines authorized for emergency use was 1.72 (30). Significantly higher values (aRR 12.61; 95% CI 5.27 – 34.47) were found when only the 18–39-year-old subjects were studied (30). However, when the two vaccines were considered separately, it was shown that in the group of patients aged 18-39 years who had received two vaccine doses the number of excess cases of MYO in the risk period was 5.7 for the Pfizer vaccine and 12.8 for the Moderna vaccine. Higher rates of MYO/pericarditis following Moderna preparation were also reported in Canada and in UK (31, 32). In Canada, it was evidenced that rates of MYO/pericarditis after dose 2 of vaccines were 28.2 per million with Moderna vaccine and 8.7 per million after Pfizer vaccine (31). In the UK, the overall reporting rate of all suspected MYO, including non-vaccine attributable causes, during the period in which mRNA vaccines were administered was 9 per million doses of Pfizer/BioNTech vaccine and 17 per million doses of Moderna vaccine (32).

Regarding general characteristics of vaccine associated MYO, a series of examples can support the overmentioned conclusions. A study carried out in Israel in which 136 cases with definite or probable MYO following Pfizer-BioNTech vaccine administration were analyzed, showed that 19 (14.0%) of the patients were diagnosed after the first and 117 (86%) after the second vaccine dose (33). Moreover, among them 91% were males and 76% aged <30 years. Finally, compared with the expected incidence, the incidence ratio of vaccine-associated MYO was 5.34 (95% CI, 4.48 to 6.40), with the highest values after the second dose in males’ recipients between the ages of 16 and 19 years (13.60; 95% CI, 9.30 - 19.20). The main risk window was the first week after the second dose. Similar data were collected in the second study carried out in Israel (34). In this case, the estimated incidence per million persons who had received at least one dose of the Pfizer-BioNTech vaccine was 21.3 cases (95% CI, 15.6 – 27.0), with the highest values (106.9 cases per million persons; 95% CI, 69.3 – 144.6) in male patients aged 16 - 29 years. The data collected through the Vaccine Adverse Event Reporting System (VAERS) of the USA further confirmed these findings (34). In this study characteristics of 1626 cases of MYO reported during the period between December 2020 and August 2021 when 192,405,448 persons received 354,100,845 mRNA-based COVID-19 vaccine doses were analyzed (35). It was shown the 82% of the cases occurred after the second dose, the median age of patients was 21 years, the median time to symptom onset 2 days, and males were 82% of the total. In detail, after the second dose the rates of MYO were highest in adolescent males aged 12 to 15 years (70.7 per million doses of the Pfizer-BioNTech), in adolescent males aged 16 to 17 years (105.9 per million doses of the BNT162b2 vaccine), and in young men aged 18 to 24 years (52.4 and 56.3 per million doses of the Pfizer-BioNTech vaccine and the Moderna vaccine, respectively) (35).

Recently collected data have increased information regarding risk of MYO in younger subjects confirming what has been previously reported for older children and adolescents and suggesting that the risk of MYO in children aged 5-11 years could be significantly lower than that reported in older subjects. The analysis of the reports to VAERS of MYO after Pfizer-BioNTech vaccination in children and adolescents aged 5–11, 12–15, and 16-17 years confirmed that even in these age groups vaccine related MYO is more common among males and occurs more frequently in the first days after the second vaccine dose. Regarding incidence, the analysis of the data collected up to December 19, 2021, regarding a total of 37,810,998 Pfizer-BioNTech vaccine doses (8,674,378 in children 5-11 years old and 18,707,169 in children 12-15 years old) has shown that rates of MYO were not substantially different, although slightly lower, from those collected in previous studies, at least for subjects older than 12 years of age (36). In particular, in adolescents aged 16-17 years incidence of MYO per million of given doses within 7 days from immunization were in males 6.1 and 70.2 after the first and the second dose, whereas they were 0.0 and 7.6 in females, respectively. In children aged 12-15 years, estimations were 4.8 and 45.7 for males and 1.0 and 3.8 for females. Finally, in children aged 5-11 years, values were 0.0 and 4.3 for males whereas, due to the low number of collected cases, in females it was possible to calculate only the incidence after the second dose that was estimated to be 2 (36).

## Mechanisms of mRNA Vaccine-Induced Myocarditis

To date, the causative relationship between COVID-19 mRNA vaccine administration and MYO development is supported only by the close temporal correlation between the 2 events. No pathophysiological mechanism capable of explaining the reason why these vaccines cause heart damage has been definitively ascertained. However, several pathogenetic factors have been suggested, although it cannot be excluded that, at least in some patients, the development of MYO is totally independent from vaccine administration (37). **Table 1** summarizes the main pathogenetic mechanisms associated with the development of MYO after vaccination with COVID-19 mRNA vaccines.

**Table 1:** Main pathogenetic mechanisms associated with the development of myocarditis after vaccination with COVID-19 mRNA vaccines.

Possible pathogenesis	Possible mechanism
mRNA vaccines as antigens causing, particularly in genetically susceptible subjects, an abnormal response of the innate and acquired immune system Superimposition of the vaccination on the immune response formed earlier as a result of the disease	Activation of natural killer lymphocytes, macrophages, and a massive cytokine release leading to a massive damage of the heart tissue Excessive immune response for the addition of the immune response to previous SARS-CoV-2 infection to immune response to mRNA vaccine
Molecular mimicry between the spike protein of SARS-CoV-2 and similar cardiac protein sequences such as α-myosin, a structural component of cells involved in heart muscle contraction	Antibodies directed against the spike protein react with heart autoantigens
Testosterone and estrogens exert opposite effects on the immune system activity.	Testosterone increases the cellular immune response favoring the pro-inflammatory effect, whereas estrogens inhibit pro-inflammatory T cells resulting in a decrease in cell-mediated responses

Development of MYO generally occurs within a week from vaccine administration. MYO is part of the clinical manifestation of COVID-19 which has an incubation period of few days. This means that a number of MYO cases seen in proximity of COVID-19 immunization could be due to an undiagnosed SARS-CoV-2 infection occurring in the same period. Among the supposed pathogenetic factors, the most accredited is the one that considers that the mRNA of the vaccines could act as an antigen causing, particularly in genetically susceptible subjects, an abnormal response of the innate and adaptive immune system, with the activation of natural killer lymphocytes, macrophages, and a massive cytokine release leading to a massive damage of the heart tissue. This supposition seems supported by the evidence that in a patient with clinical and cardiac magnetic resonance imaging (MRI) features consistent with MYO after the second mRNA vaccine administration elevated plasma levels of some immunological mediators (interleukin 1 beta receptor antagonist, interleukin 5, and interleukin 16) were observed (38). Moreover, this supposition could explain why Moderna vaccine that contains a greater amount of mRNA than the Pfizer vaccine, is associated with a higher inceidence of MYO. Contrary to this hypothesis is, however, the evidence that damage due to the abnormal immune response was never seen in other organs in which the uptake of mRNA was demonstrated (39). Moreover, it cannot be excluded that MYO following COVID-19 mRNA vaccines is secondary to the superimposition of the vaccination on the immune response formed earlier as a result of the disease. The importance of repeated exposure to coronavirus antigens seems indirectly confirmed by the fact that MYO is frequently diagnosed after a second vaccine dose. However, recently collected data regarding the development of multisystem inflammatory syndrome in children (MIS-C) after COVID-19 Pfizer-BioNTech vaccine administration seem to indicate that the risk of an abnormal immune response as cause of vaccine-related MYO is unlikely (40). In a surveillance program for MIS-C monitoring in the USA, it was reported that the rate of MIS-C after mRNA vaccine administration was extremely rare and significantly lower than that found in children infected by SARS-CoV-2, for whom an abnormal immune response is considered one of the most likely pathogenetic options (41). It was shown that, among the 21,335,331 individuals aged 12–20 years that, as of August 31, 2021, had received one or more doses of a COVID-19 vaccine, only 21 individuals had a syndrome that could be classified as MIS-C. Rate was 1.0 case per million vaccinated subjects, compared to 224 per million in children aged 11–15 years and 164 per million in those aged 16–20 years among those infected by SARS-CoV-2. Moreover, 15 MIS-C cases (71%) had evidence of SARS-CoV-2 infection casting doubts about attribution. A second potential mechanism could be represented by the molecular mimicry between the spike protein of SARS-CoV-2 and similar cardiac protein sequences such as α-myosin, a structural component of cells involved in heart muscle contraction (42). Antibodies directed against the spike protein could react with heart autoantigens and cause MYO. However, although autoantibodies have been reported in patients with MYO, their causative role has never been demonstrated (43). Finally, a role could be played by sex hormones. Testosterone and estrogens exert opposite effects on the immune system activity. Testosterone increases the cellular immune response favoring the pro-inflammatory effect, whereas estrogens inhibit pro-inflammatory T cells resulting in a decrease in cell-mediated responses (44). It seems possible that males have a natural predisposition to inflammation development, and this could explain why COVID-19 vaccine related MYO is, as all the MYO cases, regardless of their origin, more common in males than in females.

## Clinical Characteristics and Outcome of Myocarditis Associated With COVID-19 mRNA Vaccination

Acute clinical manifestations of MYO associated with mRNA vaccine administration are quite like those reported after a viral infection, including SARS-CoV-2 infection (35). The most common symptoms are chest pain, pressure or discomfort, palpitations, shortness of breath and non-specific manifestations such as fatigue. Signs suggesting MYO are tachycardias, arrythmias and, in severe cases, signs of cardiac failure (35). **Table 2** describes the main characteristics of MYO associated with COVID-19 mRNA vaccines.

**Table 2:** Main characteristics of myocarditis associated with COVID-19 mRNA vaccines.

Parameter	Characteristic
Onset	Within a week from vaccine administration
Signs and symptoms	Chest pain/pressure/discomfort; dyspnea/shortness of breath/pain with breathing; palpitations; syncope; in younger children, irritability, vomiting, poor feeding, and lethargy can occur
Diagnosis	histological definition of myocardial damage after cardiac biopsy
	MRI findings consistent with myocarditis
Other findings	Troponin level above upper limit
	Abnormal electrocardiogram or rhythm monitoring findings (ST-segment or T-wave abnormalities; paroxysmal or sustained atrial, supraventricular, or ventricular arrhythmias; atrioventricular nodal conduction delays or intraventricular conduction defects
	On echocardiogram, abnormal cardiac function or wall motion abnormalities
Clinical evolution	Mainly a mild disease that tends to solve in a few days with marginal pharmacological support
Prognosis	Favorable in short-term
Treatment	No data on long-term prognosis Control of arrythmias. Use of intravenous inotropic drugs when low cardiac output is demonstrated. Milrinone is the first choice as epinephrine and dopamine are generally prescribed to patients with hypotension and cardiogenic shock because these drugs have more chronotropic and arrhythmogenic potential.

The diagnosis of MYO is challenging because the signs and symptoms previously reported not specific and other diseases can have, especially in younger children and the elderly, similar clinical picture. The diagnosis of certainty requires the histological definition of myocardial damage after cardiac biopsy. This procedure is invasive and only rarely performed. Instead, a set of evaluations is used that add up the most common clinical symptoms with the results of some laboratory tests, ultrasound, and cardiac magnetic resonance. The most followed definition of MYO is that provided by the Centers for Diseases Control and Prevention (CDC) of the USA (37). In this case, MYO can be considered confirmed in presence of >1 among: a) chest pain/pressure/discomfort, b) dyspnea/shortness of breath/pain with breathing; c) palpitations; d) syncope or, in infants and children <12 years of age in presence of > 2 of irritability, vomiting, poor feeding, and tachypnea; e) lethargy associated with at least 1 between histopathologic confirmation of MYO or MRI findings consistent with myocarditis in the presence of troponin level above upper limit. A case is defined probable when clinical criteria previously reported are observed and > 1 finding among: a) troponin level above upper limit of normal; b) abnormal electrocardiogram or rhythm monitoring findings consistent with myocarditis (ST-segment or T-wave abnormalities; paroxysmal or sustained atrial, supraventricular, or ventricular arrhythmias; atrioventricular nodal conduction delays or intraventricular conduction defects; abnormal cardiac function or wall motion abnormalities on echocardiogram); d) MRI findings consistent with myocarditis. However, diagnosis of MYO remains complex and this clearly evidences why data regarding COVID-19 vaccine related MYO significantly vary between studies and true incidence remains undefined.

Regarding clinical evolution and prognosis of MYO following COVID-19 vaccines, available data seem to indicate that, in the short time and at least in patients aged >12 years, MYO is mainly a mild disease that tends to solve in a few days with marginal pharmacological support (35). Signs and symptoms are generally mild and rapidly solving regardless of the type of mRNA vaccine. Moreover, development of clinical manifestations is generally earlier as it occurs within few days after immunization compared to several days or weeks in case of viral infection so allowing a prompter therapeutic approach when needed. Unfortunately, no conclusion can be drawn for children aged 5-11 years. Vaccines for these subjects have been licensed for emergency use only very recently and the total number of MYO cases available for evaluation is too small to assure a valid analysis. No conclusion can also be drawn for long-term prognosis because the short follow-up time of patients, regardless of their age.

The generally favorable course is shown by the data collected in the USA between December 2020 and August 2021 in 192 405 448 individuals older than 12 years of age. Among the 817 persons younger than 30 years of age who developed MYO, it was shown that although chest pain, pressure or discomfort were reported in 89% of the cases, more relevant clinical manifestations, such as dyspnea and shortness of breath, were evidenced in only 30% (35). A significant decrease of the left ventricular ejection fraction (<50%) was demonstrated in only 12%. The same proportion of patients was considered severe enough to require treatment with immunoglobulin and glucocorticoids. Vasoactive medications and intubation or mechanical ventilation were used in only 12 and 2 patients, respectively. No case required heart transplant, extracorporeal membrane oxygenation, or a ventricular assist device. No case of death was reported. Resolution of signs and symptoms generally occurred within few days allowing a fast discharge from the hospital. These findings were confirmed by the recent CDC analysis of VAER data concerning younger children. Among the 224 patients aged 12-15 years with known outcomes, 208 (92%) recovered from symptoms at time of report and 16 (8%) mostly reported improved or resolved symptoms although ongoing physical restrictions or were still under investigation. Data regarding the 12 MYO cases diagnosed in children aged 5-11 years indicate that 8 recovered from symptoms at time of report and 4 were still recovering (36).

All these findings clearly evidence that COVID-19 related MYO is less severe that MYO due to other causes. In viral MYO, although more than 80% of patients spontaneously recover, there is a significant risk of death or heart transplantation in the first year after diagnosis (45). Data collected in adolescents have shown that up to 6% can require a heart transplant or result in mortality (10). Specifically, for MYO developed in COVID-19 patients, a systematic review of 41 studies detailing the clinical course of 42 cases revealed that about 38% of them needed vasopressor assistance and 22% required inotropic support. Moreover, five patients required each intubation and mechanical ventilation, four patients were supported by extracorporeal membrane oxygenation and eight died (46).

Despite the general good prognosis of MYO following COVID-19 vaccines, in children recently immunized that present signs and symptoms suggesting MYO should be referred to a pediatric cardiological center. Approach to suspected COVID-19 vaccine-related MYO is not substantially different from that followed for viral MYOs. Diagnosis should be supported by the use of traditional markers of cardiomyocyte lysis, including creatine kinase MB and troponins, electrocardiography, echocardiography and cardiac magnetic resonance. When possible, cardiac biopsy that can definitively confirm MYO should be performed. As arrhythmias are associated with a poor early outcome, in the early phase of MYO, monitoring for atrial or ventricular arrhythmias is essential. Low cardiac output should be treated promptly with intravenous administration of inotropic drugs, starting from milrinone. Epinephrine and dopamine are generally prescribed to patients with hypotension and cardiogenic shock because these drugs have more chronotropic and arrhythmogenic potential. Oral therapy should be initiated once the patient is beyond the acute stage of illness and shows persistent systolic dysfunction or heart failure (47). However, due to the milder clinical course compared to classic MYO and MIS-C MYO, strong pharmacologic support is only rarely required in patients with COVID-19 vaccine-related MYO. Use of non-steroidal anti-inflammatory drugs, immune globulin (IVIG), and steroids is not common (48) and remains controversial (49). A pediatric study comparing drug use in patients with viral MYO, MIS-C MYO and vaccine-related MYO has shown that intravenous immunoglobulin (IVIG) and steroids were frequently used in patients with classic MYO and MIS-C MYO but in only one and in none of the 9 patients with previous COVID-19 immunization (50). Vasopressors were most often used in patients with traditional viral MYO (42%) or MIS‐C MYO (52%) and rarely used in vaccine‐related MYO (22%), although this difference was not statistically significant.

## Myocarditis and Risk-Benefit Analysis of COVID-19 Vaccine Use

The evidence that, particularly in young adults, adolescents and older children, there is a possible risk of MYO after COVID-19 mRNA vaccine administration has raised a wide debate regarding the use of COVID-19 mRNA vaccines in these subjects. Theoretically, use of vaccine becomes mandatory if the advantage of the vaccine-induced protection against severe disease is greater than the risk of severe adverse events, including MYO. Unfortunately, a reliable risk-benefit analysis weighing protection against severe disease versus risks of vaccine-induced MYO is presently not possible as several factors essential for the analysis continue to change or are not definitively established.

Examples that follow can clearly evidence present limitations. Incidence rate of MYO, importance of age and underlying condition of the vaccinated person, type and efficacy of vaccines, and the number of administered vaccine doses should be carefully considered. However, as previously highlighted, true incidence of MYO is not definitively established. In some epidemiological studies MYO and pericarditis are considered a single disease, whereas in other studies cases with pericarditis alone are excluded. Moreover, role of age in conditioning risk of MYO development is not precisely defined. For children 5-11- year-old available data seem to indicate that the risk of MYO is marginal, and this could lead to conclude that for these children no problem of vaccine administration exists. However, the total number of vaccinated cases among which MYO incidence has been evaluated seems too small to allow definitive conclusions.

Protection induced by mRNA vaccines differs according to the virus variant. Protection against symptomatic infection due to the Delta SARS-CoV-2 variant offered by 2 doses of the Pfizer-BioNTech vaccine was found quite similar to that shown against the Alpha variant (51). On the contrary, vaccine effectiveness against Omicron variant has been reported lower than for Delta (51). Obviously, protection against MYO can significantly vary (52). The dose of mRNA vaccine included in vaccine authorized for children 5-11-year-old is lower than included in the vaccine authorized for older subjects. This could influence risk of MYO. Impact of booster doses on MYO incidence is unknown. Starting from these limitations, debatable are the conclusions reached in a study in which a stratified risk-benefit analysis of first and second doses of Pfizer-BioNTech vaccine in children 12–17-year-old by health status and history of SARS-CoV-2 infection was performed (53). The authors concluded that two doses of immunization was favorable only in girls with an underlying severe disease without previous infection. In boys with prior infection without comorbidities, even one dose carried more risks than benefits. However, this study has some limitations, first the fact that the calculated incidence of MYO after dose 2, higher than those reported in the already cited CDC evaluation, makes the benefit of vaccine very low or inexistent. Lacking reliable analysis, it should be considered that MYO following COVID-19 immunization is generally a rare and mild disease that, despite almost always hospitalized, tends to solve in few days. Moreover, it must be highlighted that importance of child vaccination for prevention of severe disease is much more important than previously thought. Children and adolescents can have a severe disease and cases of MIS-C are the best example in this regard. Moreover, immunization of these people can significantly reduce SAR-CoV-2 circulation and limit COVID-19 cases among vulnerable people. Finally, vaccine protection of older children and adolescents can reduce school absence limiting educational and social problems already emerged during lockdown, including long COVID (54, 55). Consequently, as long as the circulation of SARS-CoV-2 remains high and there are variants towards which the current vaccines are only partially effective, it seems necessary that the recommendations proposed by the CDC and followed by most health authorities around the world are maintained. In this regard, the decision of the health authorities of some countries (Norway, Taiwan and Hong Kong) that suspended the administration to adolescents of the second vaccine dose cannot be followed (56). All the children should receive 2 doses of the vaccine. A booster dose should be given to all the subjects 12-year-old and older. Considering the higher risk of MYO in subjects receiving the Moderna vaccine, Pfizer-BioNTech vaccine should be preferred, although there is no full agreement between the regulatory authorities of the different countries. In the USA, on May 20, 2022, only the Pfizer-BioNTech vaccine is authorized for children aged 5-11 years (57), whereas in the European Union (58) and in Canada (59) both the mRNA vaccines are authorized, although with slight differences. The Pfizer-BioNTech vaccine can be administered to children older than 5 years of age, whereas the Moderna vaccine is presently approved for use in individuals 6 years and older. Attention must be, however, played to children aged 5-11 years for whom true incidence of MYO is not precisely defined. In these subjects, UK recommendation suggesting the use of a two-dose schedule with the second dose of vaccine at an interval of 12 weeks could be followed. The longer interval in this age group reflects the strong evidence of high levels of protection against severe disease from the first dose, and the fact that countries with longer schedules (eight to twelve weeks) may have a lower rate of MYO after the second dose (60). In subjects that have experienced MYO/pericarditis after the first or the second vaccine dose, testing for evaluation of prior exposure to COVID-19 should be performed. If test is positive, it is likely that they are protected, and further doses should be avoided. If negative, further doses should be considered only after several weeks when detailed evaluation of heart function assures that the previous complication can be considered totally solved. However, all the subjects with a history of documented vaccine-related MYO must be followed for several years in order to diagnose early and adequately treat any unanticipated long-term complications.

## Conclusions

MYO is an adverse event that can follow the COVID-19 immunization with mRNA vaccines in few persons, particularly young adults, adolescents, and older children. It is generally a mild disease that should not modify the present recommendations for immunization with the authorized COVID-19 mRNA vaccines. Despite this, further studies are needed to evaluate presently undefined aspects of MYO development after COVID-19 vaccine administration and reduce the risk of development of this kind of vaccine complication. Together with a better definition of the true incidence of MYO and the exact role of the various factors in conditioning incidence variations, it is essential to establish long-term evolution of acute COVID-19 related MYO.

## Author Contributions

SE coordinated the study group and co-wrote the first draft of the manuscript. CC and RG substantially contributed to the content of the manuscript; AA and GR provided comments and suggested references; NP wrote the first draft of the manuscript. All the authors approved the final version of the manuscript.

## Conflict of Interest

SE: Speaker’s fees from GSK, Pfizer, Novartis, Sanofi Pasteur, MSD and Vifor in the past three years.

The remaining authors declare that the research was conducted in the absence of any commercial or financial relationships that could be construed as a potential conflict of interest.

## Publisher’s Note

All claims expressed in this article are solely those of the authors and do not necessarily represent those of their affiliated organizations, or those of the publisher, the editors and the reviewers. Any product that may be evaluated in this article, or claim that may be made by its manufacturer, is not guaranteed or endorsed by the publisher.
